# Influence of Hyrax screw position on dental movement and cortical bone: A study of finite elements

**DOI:** 10.4317/jced.56347

**Published:** 2019-12-01

**Authors:** Sandra-Liliana Gómez-Gómez, Junes-Abdul Villarraga-Ossa, Juan-Camilo Arcila-Monsalve, Diana-Marcela Moreno-Garzón, Carlos-Martin Ardila

**Affiliations:** 1 Orthodontics; Master in Epidemiology; Assistant Professor, School of Dentistry, Universidad de Antioquia; 2 Mechanic Engineer. Professor, Universidad de Antioquia; 3 Orthodontics, Universidad de Antioquia; 4 Ph.D in Epidemiology; Biomedical Stomatology Group, Universidad de Antioquia, Medellín, Colombia. Department of Periodontology, School of Dentistry, Universidad de Antioquia

## Abstract

**Background:**

Rapid maxillary expansion (RME) has effects on the dental and periodontal structures of the parts involved, which vary according to the design and position of the expansion screw. The purpose of the study was to determine the optimal three-dimensional position of the Hyrax screw to obtain precise control of the dental movement and effect on the bone cortex using the finite element method (FEM).

**Material and Methods:**

RME was performed from the patient whom two Cone-Beam computerized tomography scans (CBCT) were obtained: T1 before expansion, and T2 three months after stabilization of RME. The FEM model was designed with T1 and of Hyrax photographs. FEM was obtained by comparing the simulation, T2, and clinical results. Three sagittal screw positions (anterior-middle-posterior) and vertical (upper-medium-low) were evaluated.

**Results:**

A coronal- buccal displacement of premolars and first molars was found which decreased in the occlusal-apical direction, presenting different types of dental movement in the screw positions; besides, a tendency of translational movement in the posterior-high location was observed. In the posterior-high position a higher concentration of efforts and homogeneous deformations in the periodontal ligament and vestibular cortex of the cervical area of first molars, first and second premolars were observed, with variations according to the screw position and the distribution of stresses.

**Conclusions:**

The ideal location of the expansion screw for controlling dental movement and periodontal side effects was the high-posterior position.

** Key words:**Maxillary expansion, dental movement, finite element analysis, orthodontic appliances.

## Introduction

Rapid maxillary expansion (RME) is the most commonly used treatment for the correction of the transverse deficiencies of the upper jaw; also, it is the only one that produces a mechanical separation of the palatine bones. The Hyrax designed by Biederman (1,2) is a widely selected mechanism because it facilitates dental hygiene compared to other expanders, besides it permits to implement orthodontic treatment simultaneously (3,4). This appliance is made of stainless steel with the disjunctor screw in the mid palatine area and usually anchored to the first premolars and permanent first molars utilizing bands (5,6).

Several studies show the effects of Hyrax on the periodontal tissues of the teeth involved in the expansion, observing a decrease in the thickness of the vestibular bone table, an increase in the palatal bone table, and the presence of dehiscence in the vestibular cortex, which predispose to the formation of gingival recessions. There is also a change in the axial inclination of the support teeth, extrusion, distal rotation, and positive torque, which causes the palatal cusps to descend below the occlusal plane (4,6-9).

Various studies have determined the best position of the height of the hyrax screw with respect to the centre of resistance of the anchor molars, using the finite element method (FEM) (3,10), where it is reported that the hyrax screw must be positioned at the same level as the center of resistance of the molars, avoiding excessive vestibular movement in both apical and coronal, generating a mass movement.

The scientific literature does not currently report a consensus establishing the ideal position of the screw; various studies recommend the most posterior and superior area in the palatal vault (2,3); others suggest a position a mid-depth of the palate and in the middle of the raffe (5).

When the need for RME is determined, the clinician takes the dental impressions of the upper jaw and sends the model to the dental laboratory, leaving the design responsibility, specifically of the position of the screw, then, it is necessary to obtain standardized parameters that allow the dentist to determine which is the most appropriate position in which to place the expansion screw.

The FEM is a computational tool widely used to solve complex physical and biological problems, including research in the area of orthodontics. This method permits to calculate the stresses and deformations of the structures analyzed; it is non-invasive and precise, which provides quantitative and detailed data on the physical behavior of tissues. The application of the FEM can predict the tissue responses through the observation of the efforts created from orthodontic mechanisms applied (11). Additionally, this method allows to simulate with accuracy and reliability the effects of any system used in dental practice, and in the case of RME systems allows to simulate the different positions of the expansion screw in terms of the control of dental movement and effects on the cortical bone (3,10,12).

This study aimed to determine the optimal three-dimensional position of the Hyrax screw to obtain the most considerable control of dental movement and its effects on bone cortex using the finite element method (FEM).

## Material and Methods

This descriptive, experimental study required a patient for the creation and validation of the finite element model. The Ethics Committee of the Faculty of Dentistry of the University of Antioquia (03-2017) approved this research.

For the selection of the patient the following inclusion criteria were applied: systemically healthy, healthy oral conditions, presence of posterior crossbite (needing RME), and skeletal maturation stage between CS1-CS3, according to the Bacceti classification (13). As exclusion criteria: a history of periodontal disease, an anterior open bite of skeletal type, and history of orthodontic appliances or maxillary expansion.

A patient was selected from the Orthodontics Postgraduate program at the University of Antioquia, from which an initial T1 cone beam-computed tomography (CBCT) was obtained (before the RME treatment), and a final T2 tomography, three months after starting the RME containment stage. The tomographies were taken with standardized features, applying Cone Beam technology tomography (Sirona®Xg5-3d, Siemens® Berlin, Germany).

This study was completed in the following phases:

1. Clinical phase

To begin RME treatment, the initial clinical condition was recorded and intra, and extraoral photographs were taken; preformed stainless steel bands were adapted in the first molars and the first upper premolars. An alginate impression (Hydrogum Zhermack - Dentsply Sirona, York Pennsylvania, USA) was obtained from the maxillary arch with the bands installed; the bands were removed and transferred to the impression and later the cast was made with type III plaster (Zhermack - Dentsply Sirona, York Pennsylvania, USA). Then, the dental model was sent to the laboratory for the fabrication of the expansion device.

A 10 mm long Hyrax screw (Dentaurum® Ispringen, Germany) was used, with the following position indications: located in the palatine midline, at the level of the upper second premolars, with a vertical position at the midpoint between the total depth of the palate and the cement-enamel joint of the first upper molar. The device was then installed on the patient, with self-polymerizing glass ionomer (Ketac-Cem easy mix® 3M ESPE, Maplewood, Minnesota, USA).

The expansion protocol was carried out with activation instructions of ¼ twice a day, once in the morning and once in the afternoon (0.5mm per day) (14), at seven am and seven pm, by the previously instructed attendant, with clinical examination and registration every 7 days until the required expansion was obtained (7 mm). Once RME treatment was completed, the disjunctor screw was blocked with acrylic resin and remained in the containment stage for three months (15), performing a clinical examination every month.

At the end of the third month containment period, the Hyrax was removed, and a post-treatment tomography (T2), and a dental model of the maxillary arch were obtained, then, the following transverse measurements were acquired: inter-pit distance between first premolars, inter-pit distance of second premolars, and inter-pit distance of first molars.

2. Computer-aided design (CAD) of biological structures: upper jaw, alveolar bone, periodontal ligament, and maxillary teeth.

With the Dicom images of the initial T1 tomography, the three-dimensional CAD model of the maxilla was constructed and processed in 3DSlicer® software (open source) to obtain a cloud of points of the upper maxillary system, alveolar bone, periodontal ligament, and maxillary teeth. This cloud of points allowed the creation of solid models using the Solid Works Dassault Systèmes® software (Paris, France) that reproduced the topography of the structures, obtaining a virtual assembly of the upper jaw, alveolar bone, periodontal ligament, and maxillary teeth.

3. Computer-aided design of the Hyrax expansion appliance.

The model of the Hyrax expansion appliance was constructed using the CAD module of Solid Works Dassault Systèmes® software (Paris, France) from photographs taken of Hyrax in the patient’s study and intraoral model; these photographs were taken with a resolution of 24.2 megapixels with a Nikon D3300® camera (Tokyo, Japan) and Tokina AT-X 100mm f / 2.8 Pro D® macro lens (Tokyo, Japan). With these photographs, the elements were assembled with a spatial distribution equal to that obtained clinically in the patient.

4. Model of finite elements of the RME system

Once the assembly of the Hyrax systems and the biological structures was obtained, the simulation of the RME was executed, using the ANSYS 17.2® software (Canonsburg Pennsylvania, USA), to obtain the nodal forces and displacements. To achieve these results, a simulation with the following characteristics was used:

Mechanical properties

The mechanical properties (Young’s Module and Poisson’s Module) of the biological elements were used from those described in the literature (12,16).

For the Hyrax structures, the composition of the disjunctor screw elements and the bands was verified, using Spectrometry analysis with Bruker Qs Magellan® optical emission spectrometer (Billerica Massachusetts, USA), performed in the Spectrometry Laboratory of the University of Antioquia, which agreed with a biomedical stainless steel AISI304 (UNS S30400), from which the mechanical properties of the Hyrax used by FEM were established ( Table 1).

**Table 1:**
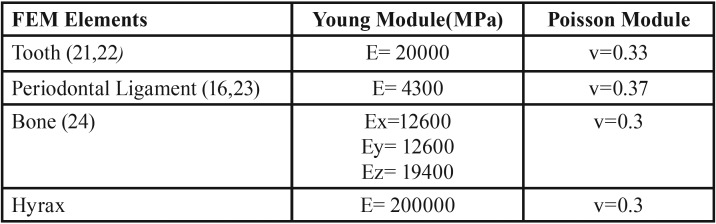
Mechanical properties of the elements for the construction of the Finite Element Model (FEM).

Mesh

**Figure 1:**
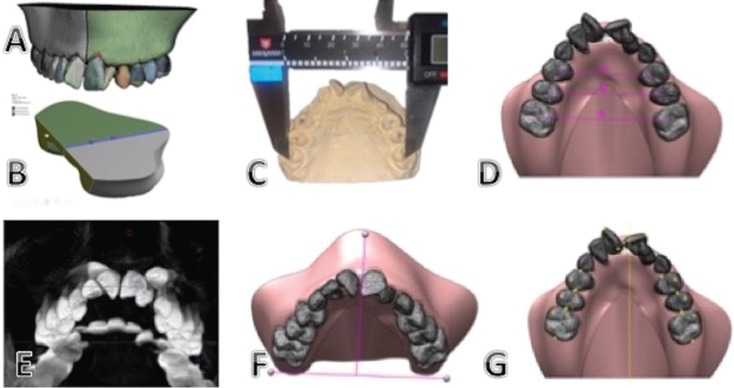
A. Finite Elements Mesh Model. B. Edge Conditions. C. Inter-pit distance measured in the dental model with a digital caliper. D. Measurements in the rapid maxillary expansion simulation. E.F. Skeletally stable points and reference lines. G. Reference points used in the T2 tomography and the simulation for the validation of the Finite Element Model.

The discretization of the system was done using tetrahedral elements of 10 nodes (pyramids with triangular base) (Fig. 1A), each component used a different number of nodes according to its volume ( Table 2).

**Table 2:**
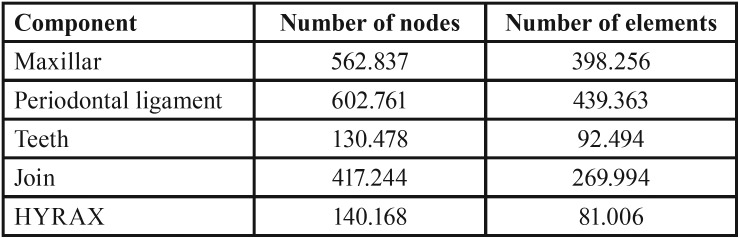
Number of nodes and elements of each component used in the simulation.

Border conditions

To accurately simulate the maxilla, displacements, and rotations on the posterior face of the model were restricted, as well as the separation lines of the maxilla (Fig. 1B). A displacement of seven mm was also applied to the central body of Hyrax to represent the displacement provided by the screw on the patient.

5. FEM validation

To validate the FEM, the data obtained in the T2 tomography, and the clinical measurements acquired from the dental movement of the six maxillary posterior teeth analyzed (first and second premolar and first molar) in the three planes of space were compared with the data obtained in the simulation with the FEM.

Comparison of clinical measures and simulation

The clinical comparison was made by measuring the inter-pit distance between first premolars, second premolars and first maxillary molars from one side to their contralateral, taken in the dental model of the patient after the treatment and the resulting inter-pit distance in the simulation (Fig. 1C,D) ( Table 3).

**Table 3:**
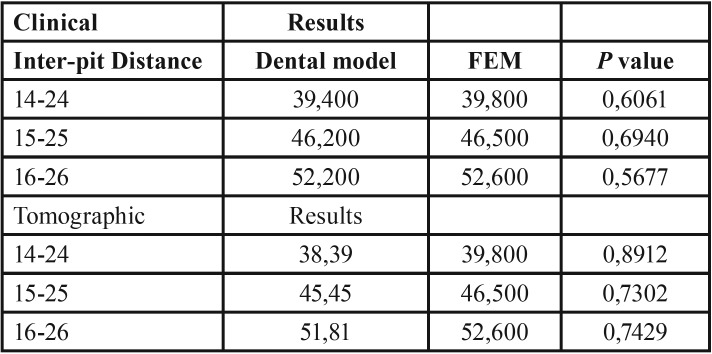
Clinical and Tomographic Results / Simulation Finite Element Model (FEM). Comparison for FEM validation.

Comparison between T2 and simulation

For the comparison between T2 and simulation, the following skeletally stable structures were selected as the reference point in T2 utilizing the Galileos viewer software (Dentsply Sirona, York Pennsylvania, USA), and transferred to the finite element model using Solid Works software (Paris, France): Anterior nasal spine (ANS), and the most distal and inferior point of the right and left pterygoid apophysis (PAR-PAL). The following reference lines were established to allow comparable measurements: PA Line: connection between PAR-PAL; ANS-PA Line: perpendicular line to PA line that passes through ANS (Fig. 1E,F).

In addition, dental reference points were established to obtain objective measurements of the changes and displacements achieved between T2 and the simulation in the maxillary arch, in the three planes of space.

**Figure 2:**
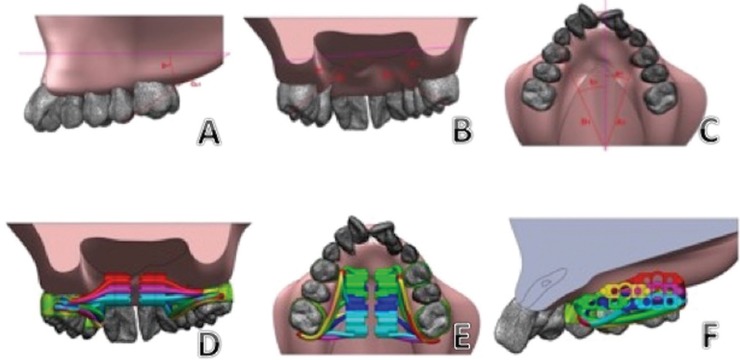
A. T2 simulation-sagittal view. B. T2 simulation-posterior view. T3 simulation-occlusal view. D. Location of the expansion screw in the vertical plane. E. Location of the expansion screw in the sagittal plane. F. Expansion screw positions in the sagittal and vertical planes.

For the sagittal and posterior views, the vertices of the vestibular cusp of the first premolars and mesovestibular cusp of permanent first molars were selected. For the occlusal view, the mesial and distal marginal ridge point of the first premolars and first permanent molars were located (Fig. 1G). With these points and reference lines, angular and longitudinal measurements were obtained for each tooth in T2 and in the simulation, in the sagittal, posterior and vertical views (Fig. 2 A-C).

6. RME simulations

Simulations were completed using the ANSYS 17.2 software of three screw positions in the vertical plane: Low: at the level of the cement-enamel joint of the upper molars. Medium: middle point between the cement-enamel joint and the maximum palatal depth. High: Maximum palatal depth (Fig. 2 D).

Additionally, simulations were performed on three screw positions in the sagittal plane. Anterior position: at first premolar level. Medium: at second premolar level. Posterior: at the level of the first permanent molar (Fig. 2 E).

The combination of these positions of the expansion screw was made; according to the anatomical limitations for its location, six models were obtained to perform the simulation: AL: anterior low, ML: medium-low, PL: posterior-low. MM: medium-medium, PM: posterior-medium. PH: posterior-high (Fig. 2 F).

## Results

1. FEM validation

Comparison of clinical measures/simulation

The comparison of the inter-pit distance obtained clinically and in the simulation was performed using a statistical test Z: difference of means with standard deviation, previously reported in the literature (25,26) with a 95% confidence level ( Table 3).

Comparison between T2 and simulation

The longitudinal measurements obtained in T2 and the simulations were compared with a confidence level of 95% ( Table 3).

The angular and longitudinal measurements were compared with the planes and reference points in the three planes of space ( Table 4).

**Table 4:**
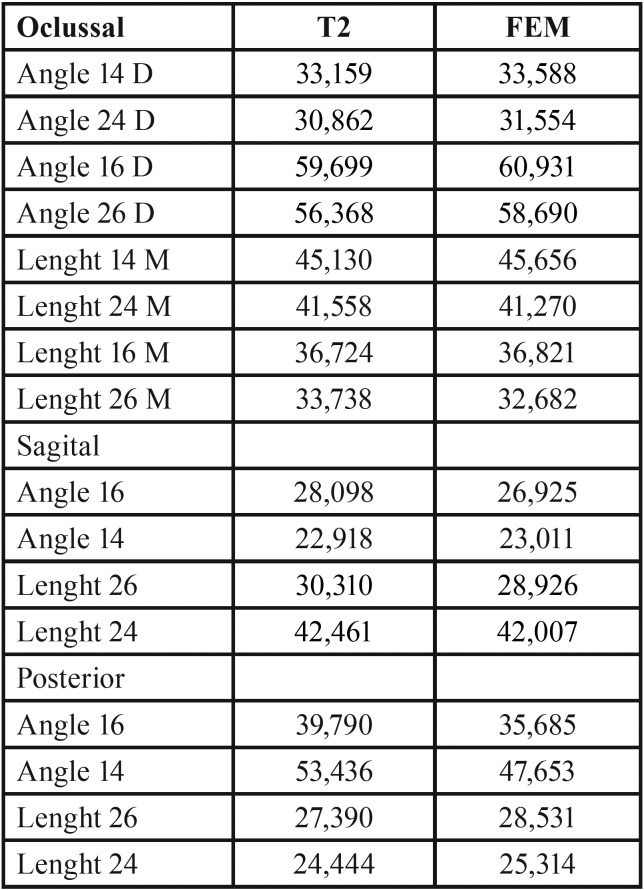
Tomographic results / Simulation. Comparison of angular and longitudinal measurements in the occlusal, sagittal and vertical planes.

Taking into account that there are no significant differences between the dental displacements among the clinical measures (tomography) and the simulation (FEM, it is concluded that the numerical method allows the simulation of the real physical phenomenon in an adjusted manner.

2. Simulation of the different positions of the expansion screw

The number of nodes and mesh elements in each model were different due to length variations in the arms of the expansion screw (data no-showed).

3. Analysis of the type of dental movement

The dental movement was evaluated according to the FEM chromatic scale, and two points were selected for each tooth: one root at the apex of the palatine root, and the other coronal (at the apex of the palatine cusp in the first and second premolar, and the mesiopalatine cusp of the first permanent molar). In each simulation, the superposition of the points was implemented and the movement in the transverse (buccal-lingual), vertical (occlusal-gingival) and sagittal (mesial-distal) planes was measured in mm (to evaluate the coronal and root movement, and from this to evaluate the tendency of the type of movement of each tooth in general).

Bucco-lingual

In all RME simulations performed by FEM, a coronal to buccal displacement was observed, which gradually decreased in occlusoapical direction.

The simulations that showed a more considerable transversal expansion at the level of first permanent molars and second premolars from highest to lowest were those corresponding to the medium-low and posterior-low models, with a tendency to till the crown towards buccal (having in common the lowest position of the expansion screw).

**Figure 3:**
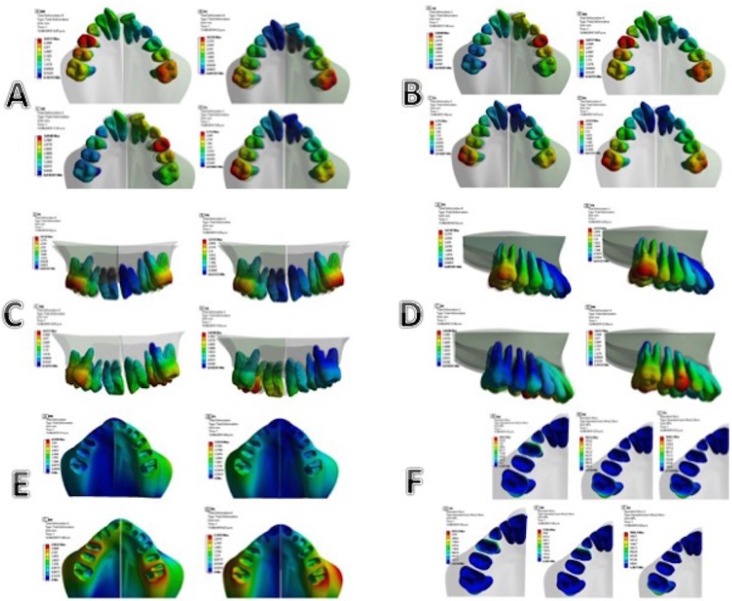
A. Type of dental movement buccal-lingual of first molars and second premolars. B. Type of dental movement buccal-lingual of first premolars. C. Type of dental movement occlusal-gingival. D. Type of dental movement mesial-distal. E. Deformation at the root/ periodontal ligament/bone interface. F. Maximum efforts at the root/ligament/bone interface.

The simulations that showed a tendency of movement with less inclination (greater tendency to bodily movement) were the models with an anterior-low and posterior-high position, which correspond to the anterior and higher position of the screw concerning the center of resistance of the molars (Fig. 3A).

The dental displacement of the first premolar with more considerable expansion and inclination was observed in the anterior-low and medium-low positions, these correspond to a sagittal location of the screw near the first premolar, and vertical distant to the center of resistance. The posterior-high and posterior-middle models showed a lower tendency of coronary inclination (Fig. 3B).

Occlusal-gingival 

In the simulations, a behavior of total movement towards apical was observed, including the first premolar, second premolar, and first permanent molar, with a tendency of more significant displacement towards apical in the posterior-low and posterior-middle models (having in common the most posterior position). On the contrary, there was a slight movement towards occlusal in the medium-low and anterior-low models, being the anterior and medium-low positions the ones that generate the most significant extrusion tendency (Fig. 3C).

Mesio-distal 

For the mesiodistal movement of the anchorage teeth, a similar behavior was observed in the first, second premolar and first permanent molar, where the simulations with posterior screw position expressed a tendency of higher distal-vestibular coronal rotation. While the tendency of rotational displacement towards mesial-vestibular was presented in the models with low-medium and low-anterior location (Fig. 3 D).

4. Analysis of the interface root/periodontal ligament/bone

Deformation at the interface root / periodontal ligament / bone

When evaluating the total deformation in the simulations by quantifying the chromatic guide of the FEM, a higher concentration in the deformation transmitted by the periodontal ligament to the bone in the area of first molars and both premolars was observed. This behavior is directly related to the anchoring function of these pieces in the RME treatment.

When comparing the simulations, more significant deformation was evidenced in the cervical and vestibular cortical area in the models with medium-low and posterior-low position, which correspond to an occlusal or low position of the expansion screw. This can be related to a more significant movement of inclination of molars, and premolars, since the force is applied distant to the center of resistance of these teeth. In the medium-medium and posterior-high models, a lower load concentration is observed in the periodontal-bone ligament interface, where the screw position is closer to the center of resistance of the anchorage pieces; generating at the same timeless effort in the vestibular table, which may be associated with less adverse events at the periodontal level (Fig. 3 E).

Efforts at the root/periodontal ligament/bone interface:

The maximum efforts in the root/ligament/bone interface were observed in the order of 5338 mPa in the medium-low simulation followed by 2653.3mPa in the anterior-low simulations located on the mesial cervical surface of the first premolar; followed by 1866.3mPa in the posterior-low simulations; which was located on the distal-lingual cervical surface of the first permanent molar; according to this, the location of the stresses is related to the location of the disjunctor screw in the antero-posterior direction, while the magnitude of the stress is related to the vertical position, with the lower position being the one that generates the greatest concentration of stress during the RME (Fig. 3 F).

## Discussion

RME and its effects have been studied applying FEM (3,12,16-19), documenting to be efficient as a tool for the simulation of the behavior of biological structures; however, these studies have as limitation the lack of methodological validation through clinical data; however, in the present study the validation FEM from a real clinical model and tomographic images provides a higher degree of reliability and accuracy of the model fit, and the results observed in each simulation.

Additionally, to ensure the reliability of the system, the spectrometry validated the type of alloy used to create the expansion device for the assignment of mechanical properties in the FEM. Besides, the FEM was refined, applying the properties of biological structures established bibliographically (12,16).

Particular authors have analyzed the influence of the position of the expansion screw in different planes of space. They evaluated the influence of the height of the expansion screw on the degree of dental inclination of molars and premolars during RME applying the FEM (3), results corroborated in the present research, where a higher tendency of buccal inclination of the crown of molars and premolars was evidenced; also, the location of the expansion screw moves away from the center of resistance of the molars in the vertical plane. On the other hand, a tendency of dental bodily movement was observed when the screw was positioned close to the center of resistance of the anchorage teeth, in the middle-middle and posterior-high positions; which may be related to the decrease in dental and periodontal effects, such as the uncontrolled crown inclination of the anchorage teeth, extrusion and descent of the palatine cusps, thinning of the vestibular bone board, and predisposition to the formation of bone dehiscence and gingival recessions (8).

Regarding the tendency of movement in the vertical plane, the results of the present study showed a tendency of intrusion movement, except with the location of the screw in the medium-low and anterior-low position, which had extrusion tendencies. It should be noted that the vertical intrusion behavior of the anchorage teeth is based on minimal displacements and is mainly related to a modification in the torque of the teeth involved, giving a change in the inclination of the crowns of the anchorage teeth than a trend of intrusion itself. Araugio *et al.* (3) found that in the position of the screw located above the center of resistance of the first permanent molar, there was a tendency to extrusion, while the positions at the level and below the center of resistance had an intrusion predisposition. It should be taken into account that the high position described by Araugio *et al.* could not be located in a real patient due to the anatomical limit of the palatal vault. The other vertical positions described have similar behavior to the present study, where an intrusion tendency of the involved teeth was observed.

Araugio *et al.* (3) reported that in the sagittal plane, a mesial displacement of the crowns was observed when the screw was located at the level and below the center of resistance, while the high position presented a tendency of coronal movement towards distal. It is relevant to consider that the antero-posterior position of these simulations was located at the level of first permanent molars, which corresponds to the posterior position of the present study, in which a similar behavior is observed in the models with the posterior location of the expansion screw. On the contrary, the model with anterior location of the expansion screw presented a tendency of coronal movement towards distal, this is related to the modification of the point of application of the force in the antero-posterior direction, generating a tendency of rotation of the crowns of the anchorage teeth towards mesial-vestibular and distal-lingual when the location is anterior, and a tendency of rotation distal-vestibular and mesial-lingual when the location is posterior.

Several authors (3,20) found that the amount of maxillary expansion is positively correlated with the magnitude of the buccal inclination of the crowns of the anchorage teeth of the expansion device. These findings are consistent with the results obtained in the present study where a greater expansion was observed due to the buccal inclination with a screw configuration below the center of resistance of the posterior teeth (observed in the anterior-low, medium-low and posterior-low positions). When the screw is located close to the center of molar and premolar resistance, the uncontrolled tilting effect of the crown can be minimized to obtain an expansion with a greater orthopedic component than dentoalveolar, which may require more treatment time to obtain the required amount of expansion.

Fernandes *et al.* (16), evaluated the tension and deformation patterns in the maxillary bone and in the mid-palatal suture, simulating different vertical and antero-posterior positions of the Hyrax expansion screw; they found a difference in the patterns of distribution of efforts, modifying the position of disjunctor screw, reporting an area of greater distribution of stresses and deformations with a screw position located at the level of the second premolars and close to the occlusal plane, similar to the findings of the present study in which the area of greater deformation and stress concentration of the root/periodontal ligament/bone interface was evidenced in the medium-low position at the level of the first premolar.

The scientific literature has reported that the dentoalveolar expansion produces a thinning of the vestibular bone table, as well as the development of bone dehiscences that tend to remain in time and predispose to the formation of gingival recessions, associated with the buccal movement of coronary inclination. The findings of these investigations suggest that applying a bodily movement of the anchorage teeth in the expansion facilitates the control and reduction of the periodontal effects in the vestibular cortical (described as the effect of dentoalveolar expansion) (7,8,9,15,20). On the other hand, Nguyen *et al.* (21) report that there is a significant recovery of the vestibular bone table in the long-term once the active treatment has been completed, mainly in the molar area and in a smaller amount in premolars. Other studies suggest the expansion through SARME (Surgically Assisted Rapid Maxillary Expansion), in cases with high periodontal risk (13,19,22,23).

The presence of external root resorption in the first premolars and first molars has been described as an adverse effect of the RME procedure. Odenrick *et al.* (24) reported that this occurrence appears mainly on the buccal surface of the vestibular roots, in the middle and cervical region; however, the presence of a partial remodeling of the root cement after six months of retention has been described (25-27). The results of this study permit to extrapolate the analysis of the stresses and deformations evaluated at the root/periodontal ligament / bone interface, in which a distribution of maximum stress concentrated in the cervical third of the anchorage teeth was observed.

The limitations of this study include the limitations of the FEM; this model is a numerical method that can present numerical and geometric approximation errors. Homogeneous and continuous materials are assumed, in addition, the edge and load conditions try to reproduce the real phenomenon, therefore, clinical validation becomes relevant; however, the results cannot be extrapolated to all patients due to anatomical variation between them, but it allows an exact approach to the behavior of the real phenomenon, which can be complemented by clinical studies (28,29).

The results of this study show that during the RME there is a vestibular inclination movement of the crown of the anchorage teeth, which decreases, in the corono-apical direction, with a greater magnitude when the disjunctor screw is located close to the occlusal plane. Therefore, these results suggest that the configuration of the expansion screw at the time of its construction must be close to the center of resistance of the anchorage teeth vertically, and in the antero-posterior direction the most posterior position. The position located at the level of the first molars was the one that presented a better distribution of stresses and deformations, with a more homogeneous distribution of efforts at the root/ligament/bone interface, and with the lowest concentration of stresses in the cervical area. It is also the position that presents a greater tendency of bodily movement of the first upper molar; this is the position that is closest to a moment / force ratio of 10/1, which according to Smith and Burstone (30) would result in a pure movement of translation, avoiding the adverse effects of the uncontrolled crown inclination (moment / force ratio of 0:1 to 5:1), in which the apex and the crown move in opposite directions, the apex generating more pressure on the palatal bone table, and the crown on the vestibular cortical.

It is relevant to emphasize that the location of the expansion screw should be selected according to the individual clinical needs of the patient, to reduce the multiple dental and periodontal adverse effects that may occur during RME treatment (4,6-9,17).

## Conclusions

1. Depending on the location of the Hyrax expander screw in the three planes of space, different trends in dental movement and periodontal tissue behavior can be obtained.

2. The location of the expansion screw in a high vertical position and posterior sagittal resulted in greater control of dental movement.

3. The posterior-high position of the expander screw offers a more homogeneous distribution of stresses and deformations at the periodontal ligament and cortical bone level, resulting in better control of the side effects of periodontal tissues.

4. The finite element method is a tool that allows simulating the behavior of the RME including the dental movement and periodontal effects.
